# Pico-Scale Digital PCR on a Super-Hydrophilic Microarray Chip for Multi-Target Detection

**DOI:** 10.3390/mi16040407

**Published:** 2025-03-30

**Authors:** Qingyue Xian, Jie Zhang, Yu Ching Wong, Yibo Gao, Qi Song, Na Xu, Weijia Wen

**Affiliations:** 1 Academy of Interdisciplinary Studies, The Hong Kong University of Science and Technology, Clear Water Bay, Kowloon 999077, Hong Kong; qxianaa@connect.ust.hk; 2 Thrust of Advanced Materials, The Hong Kong University of Science and Technology (Guangzhou), Nansha, Guangzhou 511400, China; jzhang151@connect.hkust-gz.edu.cn; 3 Department of Physics, The Hong Kong University of Science and Technology, Clear Water Bay, Kowloon 999077, Hong Kong; ycwongak@connect.ust.hk; 4 Shenzhen Shineway Biotech Co., Ltd., Futian, Shenzhen 518048, China; gyb@swtech.me (Y.G.); sq@swtech.me (Q.S.); xn@swtech.me (N.X.); 5 Zhuhai Shineway Biotech Co., Ltd., Zhuhai 519080, China

**Keywords:** dPCR, microarray, hydrophilic, POCT

## Abstract

The technology of digital polymerase chain reaction (dPCR) is rapidly evolving, yet current devices often suffer from bulkiness and cumbersome sample-loading procedures. Moreover, challenges such as droplet merging and partition size limitations impede efficiency. In this study, we present a super-hydrophilic microarray chip specifically designed for dPCR, featuring streamlined loading methods compatible with micro-electro-mechanical systems (MEMS) technology. Utilizing hydrodynamic principles, our platform enables the formation of a uniform array of 120-pL independent reaction units within a closed channel. The setup allows for rapid reactions facilitated by an efficient thermal cycler and real-time imaging. We achieved absolute quantitative detection of hepatitis B virus (HBV) plasmids at varying concentrations, alongside multiple targets, including cancer mutation gene fragments and reference genes. This work highlights the chip’s versatility and potential applications in point-of-care testing (POCT) for cancer diagnostics.

## 1. Introduction

Digital polymerase chain reaction (dPCR) is a method for the absolute quantification of a nucleotide sequence [1], playing a crucial role in biomedical researches and advancing molecular diagnostics. First introduced by K.W. Kinzler and B. Vogelstein in 1999 [2], dPCR did not gain widespread development until the advent of the first commercial instruments [3]. While dPCR is based on the same biochemical principles as quantitative real-time polymerase chain reaction (qPCR), it differs in its quantification approach. In dPCR, samples, reagents, and probes are partitioned into numerous isolated reaction units, such as microwells or droplets. Ideally, each unit contains either 0 or 1 target template, allowing for absolute quantification based on the accumulated signal during PCR amplification, regardless of the amplification efficiency and background. Compared to qPCR, the binary nature of dPCR minimizes the influence of reaction inhibitors and impurities, leading to more reliable quantitative assessments [4], particularly for detecting biomarkers in diseases such as cancer and infectious diseases. Moreover, it is an endpoint detection method that typically does not require real-time data. The partitioning in dPCR heavily relies on microfluidic technology, which includes designs for droplet generation and lithography-based methods utilizing micro-electro-mechanical systems (MEMS) to create microfluidic chips with various compartments [5]. Recent advancements in microfluidics have enabled dPCR to be performed in nanoliter or picoliter reaction units, achieving high-throughput analysis with minimal reagent consumption and a broader dynamic range of Poisson distributions [1,4,6]. Currently, dPCR technology is widely applied in single cell analysis [6,7], cancer diagnostics [8,9,10], bacteria or virus detection [11,12,13,14] as well as quantitation and preamplification of next-generation sequencing libraries [1,15,16].

Existing dPCR platforms can be categorized into two primary types: chamber-based dPCR system (cdPCR) and droplet-based dPCR (ddPCR) system, based on their partitioning methods [1]. cdPCR utilizes physical structures as partitions, while ddPCR employs a continuous oil phase to separate water, forming droplets through mechanisms like T junctions, flow-focusing or coflowing structures [6]. For instance, the Stilla Naica System for Crystal Digital PCR generates monodisperse droplets through the injection nozzles with pressurization [17]. Other notable ddPCR systems include Bio-Rad’s QX100/200 [18,19] and DPBio’s Nebula Auto Digital PCR System [20]. Although these platforms can produce tens of thousands of droplets for high-throughput analysis, droplet merging is a common issue, and ddPCR systems require constant flow and surfactants for droplet stabilization [21]. In addition, the droplet generator is separated from the reaction or detection device, adding complexity in operation.

In contrast, cdPCR systems avoid these challenges due to their physical walls, which provide mechanical and thermal stability as well as chemical resistance [6]. Meanwhile, MEMS techniques facilitate the efficient fabrication of nano/micro chambers with high replication accuracy. A typical example is Thermo Fisher’s Quantstudio 3D [22,23], which features an “open-array plate” with 20,000 etched through-holes with hydrophilic coating for capillary loading of 0.8 nL samples, followed by sealing with immiscible oil to prevent evaporation and cross-contamination. However, this kind of cdPCR systems based on open-array [21,24] suffer from sample evaporation during loading, affecting result accuracy and limiting partition unit size. Heyries, K. et al [25] presented a PDMS-based digital PCR device that employs surface tension-based sample partitioning and dehydration control to achieve high-fidelity amplification of single DNA molecules in 1,000,000 picoliter-volume reactors. Fluidigm’s BioMark system [26] features microfluidic channels and chambers equipped with flexible valves that direct the sample into 770 partitions of 0.85-nL volume. Precise distribution is achieved with high throughput and low consumption, but the numerous pipelines and nanoflex valves increase the complexity of the design. Additionally, many systems often rely on chemical modifications, such as coatings with Sigmacote [4], fluoropolymers [21], or silanes [27], to achieve desired wettability, which may not withstand high temperatures, pressures, and voltages, thereby limiting their compatibility with MEMS processes. Therefore, there is an urgent need to develop a cdPCR platform that addresses these limitations with minimal complexity.

In this study, we present a super-hydrophilic microarray chip for dPCR analysis that overcome the above limitations. We reduced the volume of reaction units for cdPCR to 120 pL, which was found to be uniform and the size can be controlled. The loading and distribution of the reaction units can be achieved at atmospheric pressure solely through hydrodynamics based on the chip surface’s wettability, eliminating the need for scraping, injection or pressurization. The super-hydrophilic surface was created by a silica layer formed inside the microwells, instead of chemical grafting, allowing it to withstand high temperatures, pressures and voltages, thus maintaining performance during subsequent anodic bonding with processed glass. Our protocol is fully compatible with MEMS technology, enhancing its potential for high-integration systems. A platform was established for dPCR reaction and detection, enabling precise quantification down to as few as 10^0^ copies/µL of plasmid DNA containing hepatitis B virus (HBV). We further investigated the detection of B-Raf proto-oncogene serine/threonine kinase (BARF) mutation gene fragments, epidermal growth factor receptor (EGFR) mutation gene fragments, and human glyceraldehyde-3-phosphate dehydrogenase (GAPDH) endogenous reference genes. A performance comparison with the commercial Naica 6-color digital PCR system demonstrated comparable stability and accuracy in detection while reducing time and operational complexity. By eliminating the need for bulky pumping systems, our proposed dPCR platform offers a user-friendly, durable and cost-effective solution for nucleic acid quantification.

## 2. Materials and Methods

### 2.1. Fabrication of the dPCR Chip

**Figure 1:**
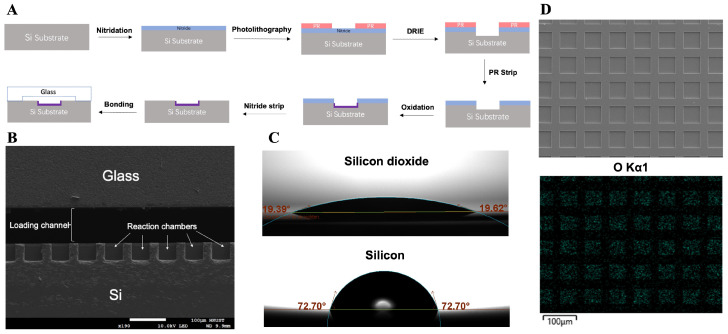
(**A**) Fabrication protocol of the super-hydrophilic microarray chip. (**B**) SEM image of the cross-section of the chip. (**C**) Contact angles of the silicon dioxide and silicon surface. (**D**) SEM image of the microarray surface and the corresponding EDX mapping result of O element.

dPCR chips were designed and fabricated using low-pressure chemical vapor deposition (LPCVD) and deep reactive ion etching (DRIE) technologies. The chip contains two channels, each of which has 11,500 independent uniform microwells formed on a silicon substrate. Each microwell measures 50 µm on each side. Detailed information on the fabrication process of the super-hydrophilic microarray chip is illustrated in Figure 1A.

**Figure 2:**
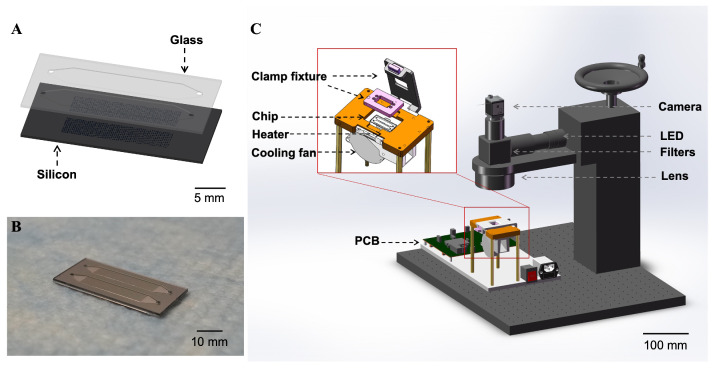
(**A**) Design of the dPCR chip. (**B**) Image of the dPCR chip. (**C**) Set up of the dPCR reaction and detection system.

Initially, a layer of silicon nitride was deposited on the silicon wafer, followed by DRIE to create the picoliter wells. The DRIE process facilitates the formation of microwells with a highly controlled vertical profile and uniform size. Subsequently, a layer of silicon dioxide was formed on the surfaces (walls and bottoms) of the microwells that were not covered by silicon nitride, employing a wet oxidation method. The silicon nitride was then removed using 85 wt % phosphoric acid at 160 °C. Finally, the silicon substrate was anodic bonded to a borosilicate glass layer that was processed to include wide loading channels and injection holes, resulting in a super-hydrophilic microwell array within a closed channel. Figure 1B presents a scanning electron microscope (SEM) image of the chip’s cross-section. The microwells have a depth and width of approximately 50 µm. Energy-dispersive X-ray spectroscopy (EDX) analysis (Figure 1D) confirms the presence of an oxide layer on the inner surfaces of the microwells. The lower image shows the mapping of the O element, where the blue dots indicate the position of O element detected. Additionally, Figure 1C illustrates the contact angle of the silicon dioxide surface, which is significantly smaller than that of the bare silicon surface, providing a super-hydrophilic surface inside the microwells. The aqueous PCR reaction solution can more easily distribute within the microwells and be separated by the oil phase due to differences in surface tension, ultimately forming independent static reaction units. Figure 2A displays the layout of a chip with two layers and Figure 2B shows the overlook of a fabricated chip.

### 2.2. Set Up of the dPCR Reaction and Detection System

The reaction solution was loaded onto the microarray, followed by the introduction of mineral oil at a flow rate of 10 µL/s to partition the reaction solution in the microwells and form independent reaction units. The inlets and outlets of the chip were then sealed with a PCR sealing film (Guangzhou Jet Bio-Filtration Co., Ltd., Guangzhou, China). Figure 2C reveals the reaction and real-time detection platform of the dPCR chip. The chip was placed on a microheater equipped with a cooling fan connected to a programmed temperature control PCB. The microheater integrated with the thermal sensor was constructed with the method presented in our previous works [24,28]. A clamp fixture was positioned atop the microheater to tightly seal the inlets and outlets of the chip, preventing liquid evaporation during thermal cycling. An optical inspection system, comprising a 470 nm LED light source and a monochrome camera (a2A1920-160umPRO, Basler, Ahrensburg, Germany), was set up for real-time observation of the reactions. Currently, the chip is for single-use only to avoid cross-contamination.

### 2.3. On-Chip Loading and Distribution

**Figure 3:**
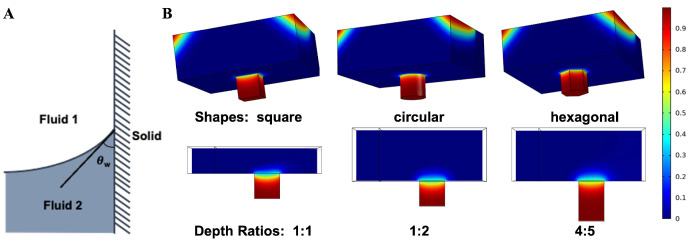
Numerical Simulation: (**A**) Schematic diagram of $\theta_{w}$ of the two-phase fluid on the wetted wall. (**B**) Volume fraction of water in different designs.

The aqueous solution for dPCR reactions was loaded to fill the entire chip under atmospheric pressure. The subsequent distribution of the aqueous solution into independent reaction units by oil is achieved through hydrodynamics, relying on the differences of surface tensions between the two phases on the hydrophilic and super-hydrophilic surfaces. The contact angles and surface tensions of water and mineral oil (Sigma-Aldrich, Inc., St. Louis, MO, USA) were measured on the surfaces of bare silicon, silicon dioxide, and glass, following standard cleaning procedures using a Theta Flow (Biolin Scientific AB, Gothenburg, Sweden). The interfacial tension between water and mineral oil was also quantified. The surface free energies of these three surfaces were calculated using contact angle data obtained with water and diiodomethane via the OWRK/Fowkes model. Using Equation (1), the interfacial energies between the liquids and solids were calculated to define the contact angles ($\theta_{w}$) of the two-phase fluid on the wetted walls (Table 1), as shown in Figure 3A. Here, $\gamma_{s1}$ and $\gamma_{s2}$ refer to the interfacial energies between the two fluids and the solid, respectively, while σ represents the interfacial tension between the fluids.

(1) $$cos\left(\theta_{w}\right)=\frac{\gamma_{s1}-\gamma_{s2}}{\sigma },$$

The parameters were incorporated into numerical simulations using COMSOL Multiphysics 5.6 to model the distribution of the aqueous phase as the oil phase was introduced. The simulation included modules for laminar flow and phase field. A time-dependent study was conducted, beginning with the loading of mineral oil at specific flow rates, with the initial state defined as completely filled with water. The surface tension coefficient of two fluids and wetted walls were defined based on the calculated results in Table 1. To optimize chip design, models with varying shapes (square, circular, hexagonal) and depth ratios of the microwells to the wide channel were tested to compare loading and distribution effects. Oil phase loading rates were varied equally to the loading of the entire microarray chip between 5 µL/s and 15 µL/s.

To assess the filling uniformity of the aqueous solution in the microwells, FAM solution was loaded and distributed on the chip. Fluorescence images of the microarray were captured, and cross-sectional images of the microarray chip with the fluorescent solution partitioned in the microwells were obtained using a confocal microscope (LSM980, Carl Zeiss AG, Jena, Germany).

### 2.4. On-Chip dPCR Analysis

#### 2.4.1. dPCR of HBV

HBV plasmid served as the target DNA to validate the quantitative detection capabilities of the designed dPCR chip. Each 10 µL PCR solution comprised 5 µL of 2× TaqMan gene expression master mix (Thermo Fisher Scientific, Waltham, MA, USA), 1 µM probe, 0.25 µM each of forward and reverse primers (GenScript Biotech Corporation, Nanjing, China), 2 µL of template solution, and nuclease-free water. HBV plasmids were prepared with $X_{dil}$ of $1\times 10^{-1}$, $5\times 10^{-2}$, $2.5\times 10^{-2}$, $1\times 10^{-2}$, $5\times 10^{-3}$, $1\times 10^{-3}$, $5\times 10^{-4}$, and $2.5\times 10^{-4}$. Amplification involved denaturation at 95 °C for 30 s, followed by 45 cycles of 95 °C for 10 s and 60 °C for 30 s. After amplification, the number of positive wells was calculated using algorithms. The sample with $X_{dil}=1\times 10^{-2}$ was also tested on the Naica 6-color digital PCR system (Stilla Technologies, Paris, France) for comparison.

#### 2.4.2. dPCR of Multiple Targets

Plasmids containing BRAF V600E and EGFR T790M mutations, along with GAPDH extracted from human sources, were detected in multiple assays using the fluorescence channels of FAM, TAMRA, and Cy5, respectively. The primers and probes (Sangon Biotech Co., Ltd., Shanghai, China) for the target DNAs were prepared (Table S1). Each 10 µL PCR reaction unit consisted of 1 µL of 10× Taq HS Buffer (Mg^2+^), 0.2 µL of dNTP mix (10 mM each), 0.2 µL of Taq HS DNA polymerase (5 U/µL, Vazyme Biotech Co., Ltd., Nanjing, China), 0.4 µM of forward and reverse primers, and 0.2 µM of probes for each target, along with a specific amount of templates and nuclease-free water. The reaction was initiated at 95 °C for 30 s, followed by 45 thermocycles at 95 °C for 15 s and 60 °C for 45 s. Fluorescence images of the dPCR results were captured using a Nikon Eclipse Ni system for each fluorescence channel.

In the multiple dPCR experiment, the 4.4 µL template solution included 1.6 µL of BRAF plasmid, 0.8 µL of EGFR plasmid, and 2 µL of human genes. Samples containing only a single template (ST) and no template control (NTC) were also tested in parallel to establish positive thresholds and investigate nonspecific amplification. The same samples were tested on the Naica 6-color digital PCR system for reference.

## 3. Results

### 3.1. On-Chip Loading and Distribution

The volume fraction of water after distribution in different designs was determined through numerical simulations of the two-phase flow (Figure 3B). Table 2 presents the calculated filling rates of water based on the collected data. Among the geometrical designs tested, square microwells demonstrated superior performance compared to circular and hexagonal shapes, leading to their selection for subsequent experiments. Among the square designs, that with a 4:5 depth ratio (80-µm microwells and 100-µm wide channels) had most water remained in the microwells after distribution. As indicated in Figure 3B, the two-phase interface is in a concave state equilibrium. Since $\theta_{w}$ is the same, theoretically, when the flow rate and wide channel depth are the same, the concave distance of the two-phase interface is also the same. Therefore, regardless of the depth of the microwell, the volume of water replaced by oil within the microwell is the same. Because the volume of a deep well is larger, the filling rate obtained in a deeper microwell increases. Considering the desired volume of reaction unit, we adopted the 1:2 depth ratio design, with microwells having a depth of 50 µm and a width of 50 µm. Additionally, we observed that decreasing the loading velocity of the mineral oil during distribution (equally to loading the entire microarray chip at 15 µL/s, 10 µL/s and 5 µL/s) resulted in more water being retained in the microwells.

**Figure 4:**
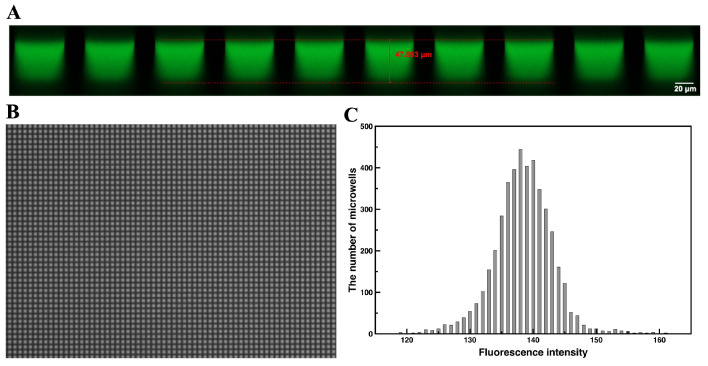
Loading and dirstribution of FAM solution: (**A**) Cross section of the microarray chip with the FAM solution patitioned in the microwells. (**B**) Fluorescence image of the FAM solution distributed on the microarray. (**C**) Histogram of the fluorescence intensity of each microwell.

To assess partition conditions and uniformity, we employed FAM as a fluorescent dye. The filling condition of the aqueous fluorescent solution in the microwells (Figure 4A) closely matched the simulation results. Based on both simulation and experimental findings, the partition volume was defined as 120 pL. If the reaction solution is evenly partitioned, the volume should remain consistent across all microwells. Figure 4B illustrates the solution distribution in the microarray chip following loading. Meanwhile, Figure 4C presents a histogram depicting the fluorescence intensity of each microwell. Some microwells showed higher or lower intensity, which is expected given the variation in light intensity between the center and edges of the optical field, particularly in large views. The average fluorescence intensity across all microwells was approximately 138, with a relative standard deviation of 3.37%. This indicates an acceptable uniformity of fluorescence intensity among the microwells, affirming that the partition volume in each microwell can be considered consistent.

### 3.2. On-Chip Thermal Performance

**Figure 5:**
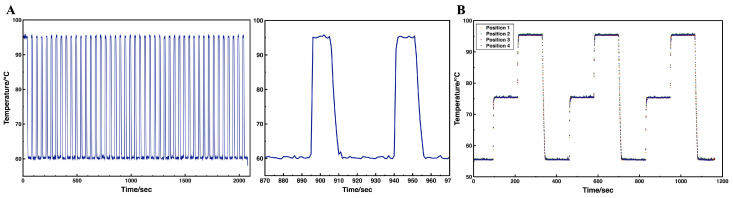
(**A**) PCR temperature profile of 45 cycles on the platform (left). Scatter diagram showing temperature pattern of two cycles (right). (**B**) Temperature uniformity of the microheater during gradient heating cycles.

Precise temperature control and uniform thermal distribution are crucial for cdPCR. Since the temperature can be rapidly reached and maintained in the small reaction unit, the robust on-chip thermal performance avoids the reduction in detection performance caused by prolonged high or low temperatures due to slow temperature ramping. The on-chip heating and temperature sensing was achieved by thin-film resistors, a sputtering patterned layer of platinum on silicon substrate. The platinum sensor effectively reflected temperature changes, enabling control over the heating process. Temperature data were recorded during thermal cycling. As illustrated in the temperature profile in Figure 5A, the set temperatures were rapidly reached and maintained throughout the cycling process. The average heating rate exceeded 17.5 °C/s, while cooling rates were approximately 5.8 to 7 °C/s, comparable to other cdPCR designs and higher than many commercial rapid PCR devices [29]. The entire program for running the HBV dPCR was completed in 36 min, which was only 30% of the reaction time required by the Naica system. In addition, to validate temperature accuracy, an external temperature sensor was used to measure the surface temperature of the heater during a gradient heating from 55 °C to 95 °C. Deviations compared with the set temperature during the plateau phase were observed to be 0.1 °C at both 55 °C and 75 °C, and 0.2 °C at 95 °C. Additionally, the heater exhibited homogeneous thermal distribution, with only a 0.1 °C difference at 55 °C and 75 °C, 0.2 °C difference at 95 °C when tested across four different positions on the microheater (Figure 5B). Since the microheater is reusable and integrated with a clamp fixture, the operator only needs to replace the chip during use, making it user-friendly and a suitable choice for POCT.

### 3.3. On-Chip dPCR Analysis

#### 3.3.1. dPCR of HBV

To validate the stability of the static reaction units on the array throughout the PCR thermal cycle, fluorescence images of the microarray were captured at the end of each cycle (Figure S1). No movement or evaporation of the reaction units was observed. Some wells at the edge of the channel were excluded from calculations due to reflections from the glass wall, as well as those not filled with the reaction solution because of fabrication defects or unstable flow at the inlets. A total of up to 10,100 wells were analyzed and classified as positive or negative.

**Figure 6:**
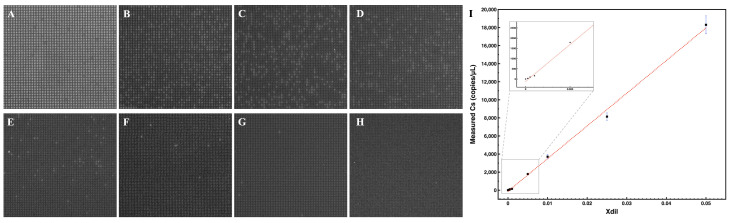
(**A**–**H**) Flourescence images of the results of the dPCR to detect different concentrations of HBV plasmids: the HBV plasmid dilution factors were (**A**) $1\times 10^{-1}$; (**B**) $5\times 10^{-2}$; (**C**) $2.5\times 10^{-2}$; (**D**) $1\times 10^{-2}$; (**E**) $5\times 10^{-3}$; (**F**) $1\times 10^{-3}$; (**G**) $5\times 10^{-4}$; (**H**) no template control. (**I**) Linear correlation between the measured DNA concentration and the $X_{dil}$. Error bars are SD. for n = 3.

Fluorescence images of the dPCR results for detecting different concentrations of HBV plasmid are shown in Figure 6A–H. As the dilution factor ($X_{dil}$) of the sample decreased, fewer positive wells were observed. The numbers of positive and negative microwells were calculated using algorithms in MATLAB (https://ww2.mathworks.cn/products/matlab.html, accessed on 4 March 2025). Ultimately, the copy number of target DNA in each sample ($C_{s}$) was deduced using Poisson’s distribution, based on the equation below [1]:

(2) $$-ln\left(1-f_{0}\right)=\frac{M}{N}=CV_{p},$$

where $f_{0}$ was the fraction of positive units over the number of partitions, *M* was the copy number of target DNA, *N* was the total number of partitions, *C* was the copy number concentration in the reaction, and $V_{p}$ was the partition volume. In the experiment, $V_{p}$ was 120 pL, allowing for the calculation of *C*. The relationship is given by $C_{s}=5C$ based on the reaction components. The linear correlation between $C_{s}$ and $X_{dil}$ is depicted in Figure 6I, where R^2^ > 0.99. The detection limit was 6 copies/µL, with a 95% statistical confidence intervals, [2.1022, 10.4154].

The measured $C_{s}$ for the sample $X_{dil}$ = $1\times 10^{-2}$ was 3708 copies/µL, closely aligning with the result of 3806 copies/µL obtained from the Naica 6-color digital PCR system, thus validating the system’s reliability. Furthermore, the results exhibited a dynamic linear range of detection from 10^4^ to 10^0^ copies/µL.

#### 3.3.2. dPCR of Multiple Targets

**Figure 7:**
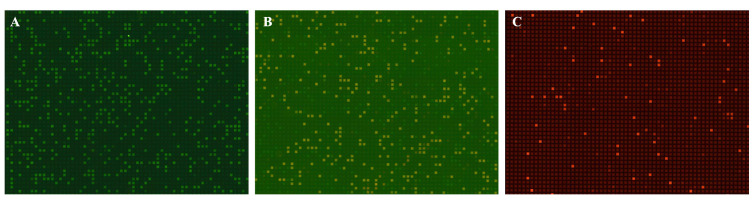
Fluorescence images of the multiple dPCR results: (**A**) BRAF (FAM). (**B**) EGFR (TAMRA). (**C**) GAPDH (Cy5).

Fluorescence images of the multiple dPCR results are presented in Figure 7, where the number of positive units can be identified. The copy number concentrations of the original samples were calculated using the previously described method, as shown in Table 3. The measured concentrations of the analytes on the microarray chip were closely aligned with those obtained from the Naica dPCR system. Minor differences may be attributed to sampling variance and variations in reaction components. Nonspecific amplification was not observed in the FAM and TAMRA channels, but small copy numbers were detected in the Cy5 channel even among its negative controls. This is acceptable, as the experimental environment was inevitably contaminated with human samples containing GAPDH endogenous reference genes. Overall, the microarray dPCR chip demonstrated reliability for multiple dPCR analyses.

## 4. Discussion

This study addresses the existing gaps in open-array cdPCR designs by presenting a closed-channel cdPCR microarray. Many current closed-channel cdPCR devices are based on polydimethylsiloxane (PDMS) [25]. PDMS PCR chips suffer from over 50% solution evaporation rates during thermal cycles because of its air permeability [30], leading to many works dedicated to address this issue, but certain evaporation rates still exist [31,32,33]. Our chip eliminated this problem by using glass as the cover slide, which is waterproof and provides high optical transmission, and high hydrophilicity. Moreover, the mechanical strength of glass wall and strong anodic bonding strength between glass and silicon provide robust physical support to stabilize the reaction units formed during the chip transfer and thermal cycling, which avoids cross-contamination among the reaction units.

Compared to existing chemical modification methods that improve hydrophilicity, our dPCR chip employed a method without chemical grafting, resulting in a stable super-hydrophilic layer that can sustain the subsequent anodic bonding process, and other processes where high temperature and voltage is applied. Therefore, the prepared super-hydrophilic array was fully compatible with MEMS technology, supporting the chip’s potential for highly integrated applications.

This design simplifies sample loading and distribution, eliminating the need for manual scraping, robotic injection, or programmatic pressure sampling, thus streamlining system construction compared to its similar designs [4,24]. Since the inner surface of the entire chamber is hydrophilic, the aqueous solution for dPCR reactions can fill the entire chip including all microwells at atmospheric pressure via continuous pipetting. The subsequent distribution into independent reaction units by oil is achieved through hydrodynamics, relying on the chip’s hydrophilicity and super-hydrophilicity, without the usage of bulk-pump.

Both simulation and experimental results confirm that the aqueous solution partitions uniformly within the microwells, exhibiting stable performance. The volume of the reaction units was approximately 65% less than most current cdPCR devices, normally 0.34–33 nL [1,4,34], which not only improves the detection sensitivity and accuracy, but also requires less reagents and samples. The rapid and uniform thermal cycles demonstrated reliable and efficient heating and cooling characteristics of the platform, which supports time-efficient reaction with the reaction time far less than commercial dPCR devices. Additionally, this platform enables real-time monitoring during the reaction, providing flexibility for various research applications.

The dPCR analyses of HBV across different dilution factors validated the method’s reliability, demonstrated by the strong linear correlation between measured concentrations and dilution factors. The detection range (from $10^{4}$ to $10^{0}$ copies/µL) compares favorably to existing works, while requiring fewer partitions. The detection limit and sensitivity for quantifying DNA can be further enhanced by increasing the number of wells or reducing the chamber volume. Thus, the method presented here can adapt to various research and clinical needs with minor modifications.

Furthermore, BRAF and EGFR mutations are critical for optimizing targeted therapies in cancer patients [35,36]. Mutations in BRAF V600E and EGFR T790M significantly influence tumor progression and resistance to treatment, particularly in non-small cell lung cancer and colorectal cancer. Identifying these mutations can guide personalized treatment strategies and improve patient outcomes. By allowing for concurrent detection of these mutations alongside GAPDH reference genes, our platform highlights its clinical relevance and potential to improve personalized treatment strategies. The results obtained demonstrated no significant differences when compared to current commercial dPCR devices, affirming the compatibility of the super-hydrophilic microarray dPCR platform with diverse reaction systems and sample types, including plasmid and linear DNAs. Furthermore, this system can be easily upgraded to support 5/6-color detection with appropriate filters and analysis algorithms, further exemplifying its versatility for diverse needs.

In conclusion, our approach emphasizes the interdisciplinary nature of modern diagnostics by bridging microfluidic technology and molecular detection. The innovation of this work spans from manufacturing to the miniaturization of reaction units, and the simplification of operations. By facilitating accurate biomarker quantification and supporting multiplex detection capabilities, this system stands to enhance POCT applications, contributing to timely and effective disease monitoring and management.

## 5. Conclusions

In this study, we developed a dPCR platform utilizing a super-hydrophilic microarray chip equipped with a real-time monitoring system. Each channel can generate 10,100 independent reaction units, each with a volume of 120 pL and the reactions of two samples was allowed on a single chip. The sample loading and distribution require no specialized equipment and can be completed in just one minute. By using mineral oil to cover the reaction samples, we enabled the reactions to be conducted at atmospheric pressure, employing a clamp fixture to seal the inlets and outlets of the channels. This setup effectively prevented evaporation and cross-contamination among the reaction units. Meanwhile, the platform’s reliable and efficient thermal characteristics significantly shortened the reaction time. We validated the dPCR system using gradient concentrations of HBV plasmid, $X_{dil}$ ranging from $1\times 10^{-1}$ to $2.5\times 10^{-4}$ as DNA templates. The statistical results demonstrated that our dPCR system can achieve absolute quantitative detection of target DNA. Furthermore, this system is capable of detecting both single and multiple targets simultaneously, showing excellent performance in the combined detection of EGFR, BRAF, and GAPDH. This highlights the compatibility of our system with diverse reaction environments and template types, including circular and linear DNAs. Overall, this dPCR device, featuring MEMS-compatible fabrication and straightforward operation, has significant potential as a powerful POCT solution in pathogen detection and infectious disease monitoring.

## Figures and Tables

**Table 1:** Parameters of the surface porperties.

Parameters	Values		
	Silicon	Silicon Dioxide	Glass
σ ^1^ (mN/m)	36.3636		
$\sigma_{s}$ ^2^ (mN/m)	50.66	73.77	60.94
$\gamma_{water-solid}$ ^3^ (mN/m)	24.620	31.361	14.119
$\gamma_{oil-solid}$ ^3^ (mN/m)	29.056	52.818	39.375
θ ^4^ (rad)	1.4485	0.9396	0.8030

^1^ σ: The interfacial tension coefficient between water and mineral oil. ^2^ $\sigma_{s}$: The surface free energy of the solid. ^3^ γ: The interfacial energies between fluid and solid. ^4^ θ: The contact angles of the two-phase fluid on the wetted walls.

**Table 2:** Filling rate of water in different designs.

Designs	Shapes			Depth Ratios			Velocities		
	Square	Circular	Hexagonal	1:1	1:2	4:5	High	Middle	Low
Filling rates (%)	96.7338	96.7120	96.0452	92.82	96.7338	98.6455	96.7338	96.7674	97.4326

**Table 3:** Measured concentration (copies/µL) of the samples in three channels.

Sample Names	FAM	TAMRA	Cy5
Multiple templates	13,031	14,104	2273
BRAF ST	12,494	0	6
EGFR ST	0	14,495	15
GAPDH ST	0	0	2280
NTC	0	0	17
Multiple templates on Naica system	13,616	15,083	2003

## Data Availability

The data that support the findings of this study are available from the corresponding author upon reasonable request.

## Supplementary Materials

The following supporting information can be downloaded at: https://www.mdpi.com/article/10.3390/mi16040407/s1, Figure S1: Fluorescence images obtained during the thermal cycling testing with HBV plasmid $X_{dil}$ = $5\times 10^{-3}$; Figure S2: Results of fluorescence images in each channel of the multiple dPCR testing with ST and NTC; Table S1: Sequences of primers and probes used in this study to test the dPCR chips; Table S2: Sequences of DNA synthesized in plasmids used in this study to test the dPCR chips.

## Abbreviations

The following abbreviations are used in this manuscript:

dPCR	Digital polymerase chain reaction
qPCR	Quantitative real-time polymerase chain reaction
MEMS	Micro-electro-mechanical systems
cdPCR	Chamber-based digital polymerase chain reaction
ddPCR	Droplet-based digital polymerase chain reaction
HBV	Hepatitis B virus
BRAF	B-Raf proto-oncogene, serine/threonine kinase
EGFR	Epidermal growth factor receptor
GAPDH	Glyceraldehyde-3-phosphate dehydrogenase
FAM	5-Carboxyfluorescein
TAMARA	5-Carboxytetramethylrhodamine
Cy5	Invitrogen Cyanine5
LPCVD	Low pressure chemical vapor deposition
DRIE	Deep reactive ion etching
SEM	Scanning electron microscope
EDX	Energy-dispersive X-ray spectroscopy
ST	Single template
NTC	No template control
PDMS	Polydimethylsiloxane
POCT	Point-of-care testing
