# Supplementary material for: Pico-Scale Digital PCR on a Super-Hydrophilic Microarray Chip for Multi-Target Detection
Source: Micromachines (Basel). 2025 Mar 30;16(4):407. doi: 10.3390/mi16040407 (PMC12029596; doi:10.3390/mi16040407)
Supplement: Supplementary file 1 [file micromachines-16-00407-s001.zip › micromachines-3539994-supplementary.pdf]

Pico-Scale Digital PCR on a Super-Hydrophilic Microarray Chip for Multi-Target Detection

Supplementary Materials

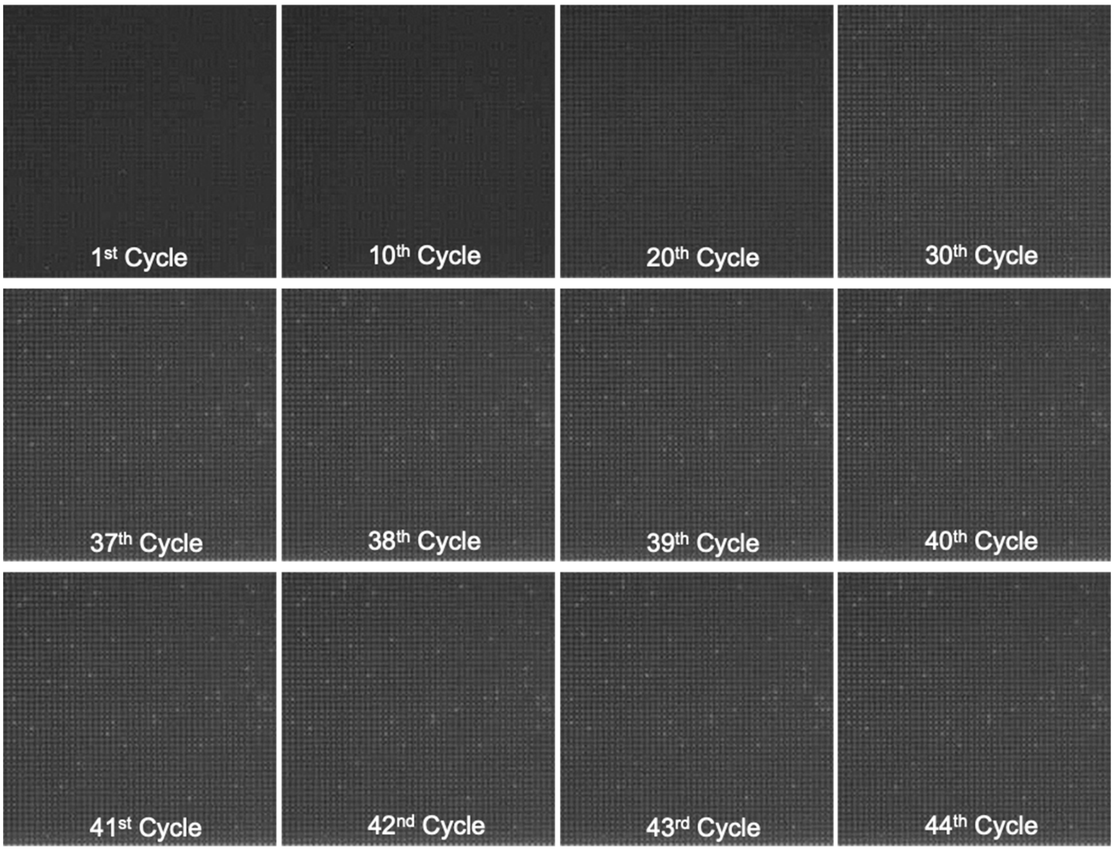

Figure S1. Fluorescence images obtained during the thermal cycling testing with HBV plasmid $X_{dil}=5\times10^{-3}$

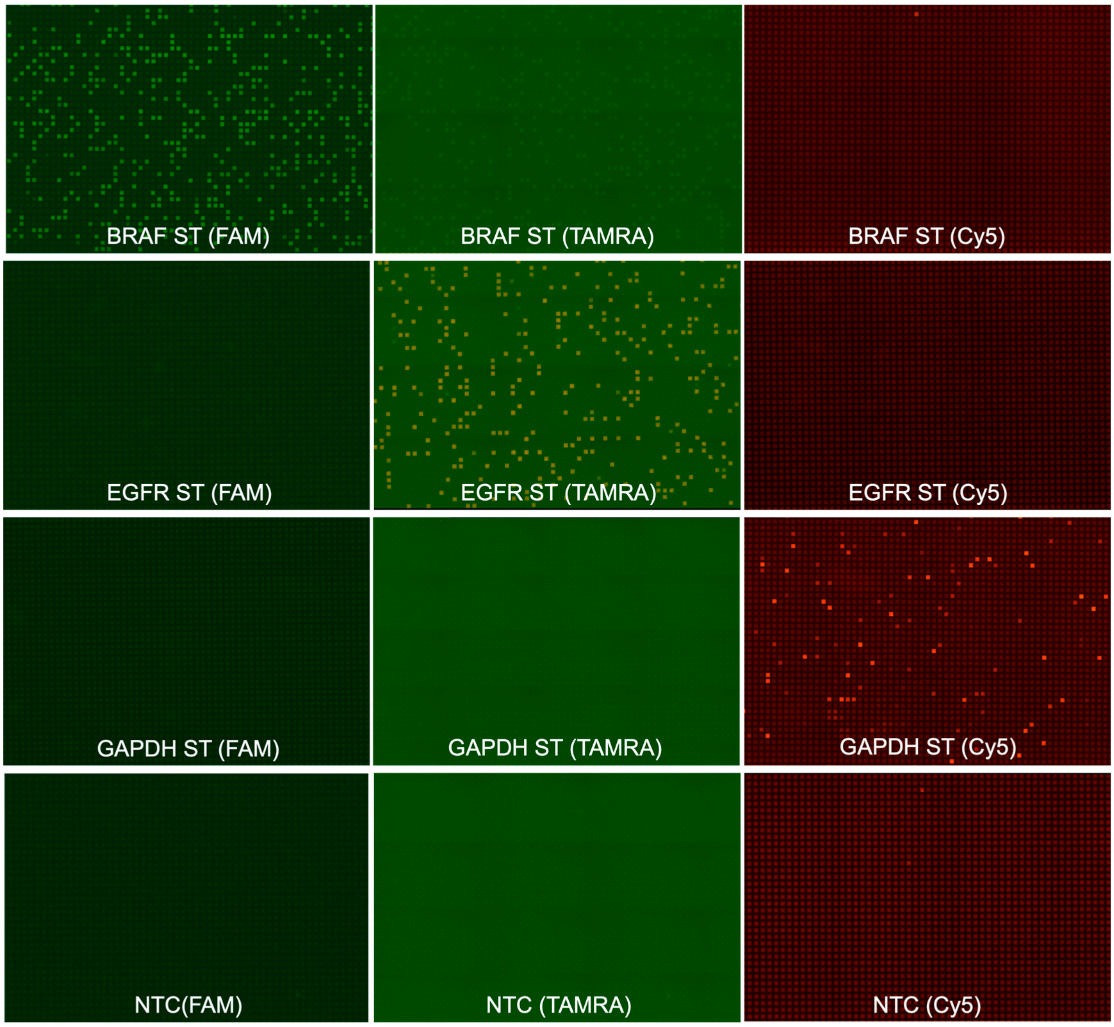

Figure S2. Results of fluorescence images in each channel of the multiple dPCR testing with ST and NTC

Table S1. Sequences of primers and probes used in this study to test the dPCR chips.

Primers and probes	Sequence (5' to 3')
HBV-2 F382	ACTCCTCCAGCTTATAGACCACCA
HBV-2 R518	CGACGCGGCGATTGAGA
HBV-2 Pb478	FAM-ACTCCCTCGCCTCGCAGACGA-BHQ-1
BRAF V600E F2R	AGGTGATTTTGGTCTAGCTACAAA
BARF V600E R	TCCAGACAACTGTTCAAACTG
BARF V600E PbR	FAM-TGGGACCCACTCCATCGAGATTTC-BHQ-1
EGFR T790M F2m	CCACCGTGCAGCTCATCTT
EGFR T790M R	CACACCAGTTGAGCAGGTAC
EGFR T790M Pb	TAMRA-CCTTCGGCTGCCTCCTGGACTATGT-BHQ-1
GAPDH F	ACTGAGCACCAGGTGGTCT
GAPDH R	ATGAGCTTGACAAAGTGGTCG
GAPDH Pb	Cy5-CAGCGACACCCACTCCTCCACCTT-BHQ-1

Table S2. Sequences of DNA synthesized in plasmids used in this study to test the dPCR chips.

Target names	Sequence
HBV-2	TCTTTCGGAGTGTGGATTCGCACTCCTCCAGCTTATAGACCACCAAATGCCCC TATCTTATCAACACTTCCGGAGACTACTGTTGTTAGACGACGAGGCAGGTCCC CTAGAAGAAGAACTCCCTCGCCTCGCAGACGAAGGTCTCAATCGCCGCGTCG CAGAAGGTCTCAATCTCGGGAATCTCAATGTTAGTATACCTTG
BARF V600E	TCTGATAGGAAAATGAGATCTACTGTTTTCCTTTACTTACTACACCTCAGATATA TTTCTTCATGAAGACCTCACAGTAAAAATAGGTGATTTTGGTCTAGCTACAGAG AAATCTCGATGGAGTGGGTCCCATCAGTTTGAACAGTTGTCTGGATCCATTTTG TGGATGGTAAGAATTGAGGCTATTTTTCCACTGATTAAATTTTTGGCCCTGAGAT GCTGCTGAGTTACTAGAAAGTCATTGAAGGTCTCAACTATAGTATTTTCATAGTT CCCA
EGFR T790M	CCCCACGTGTGCCGCCTGCTGGGCATCTGCCTCACCTCCACCGTGCAGCTCATC ATGCAGCTCATGCCCTTCGGCTGCCTCCTGGACTATGTCCGGGAACACAAAGA CAATATTGGCTCCCAGTACCTGCTCAACTGGTGTGTGCAGATCGCAAAG
